# Physical therapy and exercise in osteoarthritis prevention

**DOI:** 10.1186/1471-2474-16-S1-S14

**Published:** 2015-12-01

**Authors:** Maria Stokes

**Affiliations:** 1 Faculty of Health Sciences, University of Southampton, 104 Burgess Rd, Southampton SO17 1BJ, UK; 2 Arthritis Research UK Centre for Sport, Exercise and Osteoarthritis, C floor West Block, Queenâ€™s Medical Centre, Derby Road, Nottingham, NG7 2UH, UK

## 

Exercise encompasses physical activity (habitual, sporting), and exercise programmes to improve and maintain joint heath. Clinical guidelines recommend exercise for osteoarthritis (OA) e.g. NICE, EULAR, OARSI. Benefits of moderate exercise include weight control (obesity is a known risk factor for OA) and joint health (beneficial for cartilage). Specific exercises aim to achieve optimal biomechanics to protect joints (joint alignment, load reducing strategies) and improve muscle strength, endurance, power, flexibility and co-ordination. Other physiotherapy principles include sport/task specific exercises, personalised medicine (exercises tailored for the individual) and neuromuscular control of movement (screening and retraining using specific exercises). Manual therapy techniques (pain management, mobilisations, muscle stretching) can improve exercise outcomes. Effects of exercise on pain and function are comparable with those for non-steroidal anti-inflammatory drugs. Exercise forms part of biopsychosocial management, using tailored, patient centred interventions based on assessment, with shared decision making.

Three levels of prevention include: primary (preventing injury and onset of OA), secondary (preventing progression of OA) and tertiary (managing complicated, long-term health problems). The Arthritis Research UK Centre for Sport, Exercise and Osteoarthritis is focussing on secondary prevention of progression of injury and/or overuse to OA. Biomechanical and neurophysiological mechanisms of abnormal movement and joint loading are investigated using 3D motion analysis and electromyographic techniques, and evidence is emerging that mechanistic-based exercises can correct abnormal movement.

Movement screening tools in clinical/field environments are used increasingly to assess movement control and functional performance, primarily in sport, to predict injury and/or inform intervention. Robustness of screening tools is variable, in terms of reliability, validity and prediction of injury risk. Consensus is needed for terminology and establishing which screening tests are appropriate for specific cohorts and movement problems.

High quality longitudinal trails are needed to ensure effective use of exercise for OA prevention. Activity needs to be maintained for long-lasting effects but adherence to changing lifestyle remains a major challenge. For exercises targeting movement problems, studies need to elucidate which elements, modes, doses, and frequency and duration of exercise are optimal for specific joints and body regions.

It remains unknown whether exercise can influence disease parthenogenesis and progression. Evidence of cost-effectiveness of exercise as a clinical intervention for OA is also needed. Understanding and overcoming barriers to exercise and enabling access will be crucial for widespread uptake of exercise. Translation research is needed to determine how to change practice and influence GP referral to exercise programmes.

